# Genome-Wide Identification of the *SAUR* Gene Family in Wax Gourd (*Benincasa hispida*) and Functional Characterization of *BhSAUR60* during Fruit Development

**DOI:** 10.3390/ijms232214021

**Published:** 2022-11-14

**Authors:** Chen Luo, Jinqiang Yan, Changxia He, Wenrui Liu, Dasen Xie, Biao Jiang

**Affiliations:** 1 Vegetable Research Institute, Guangdong Academy of Agricultural Sciences, Guangzhou 510640, China; 2 Guangdong Key Laboratory for New Technology Research of Vegetables, Guangzhou 510640, China

**Keywords:** wax gourd, genome-wide identification, *SAUR*, gene family, fruit development

## Abstract

The wax gourd (*Benincasa hispida*) is an important vegetable crop whose fruits contain nutrients and metabolites. *Small auxin upregulated RNA* (*SAUR*) genes constitute the largest early auxin-responsive gene family and regulate various biological processes in plants, although this gene family has not been studied in the wax gourd. Here, we performed genome-wide identification of the *SAUR* gene family in wax gourds and analyzed their syntenic and phylogenetic relationships, gene structures, conserved motifs, *cis*-acting elements, and expression patterns. A total of 68 *SAUR* (*BhSAUR*) genes were identified, which were distributed on nine chromosomes with 41 genes in two clusters. More than half of the *BhSAUR* genes were derived from tandem duplication events. The BhSAUR proteins were classified into seven subfamilies. *BhSAUR* gene promoters contained *cis*-acting elements involved in plant hormone and environmental signal responses. Further expression profiles showed that *BhSAUR* genes displayed different expression patterns. *BhSAUR60* was highly expressed in fruits, and overexpression led to longer fruits in *Arabidopsis*. In addition, the plants with overexpression displayed longer floral organs and wavy stems. In conclusion, our results provide a systematic analysis of the wax gourd *SAUR* gene family and facilitate the functional study of *BhSAUR60* during wax gourd fruit development.

## 1. Introduction

The wax gourd (*Benincasa hispida* (Thunb.) Cogn., 2n = 2*x* = 24) is an important vegetable crop of the Cucurbitaceae family [1,2]. The fruits of wax gourds contain nutrients and metabolites, and can be used as both a food and a medicine [3]. There are several characteristics of these fruits, such as the size, shape, and peel color, which are important agronomic traits in breeding [4,5,6,7,8,9]. Previously, quantitative trait locus (QTL) mapping identified size-associated traits in wax gourd fruits, including fruit weight, fruit length, fruit diameter, as well as flesh thickness [5]. The fine-mapping results showed that *Benincasa hispida Fruit Shape* (*BFS*) was the candidate gene for wax gourd fruit shape determination [4]. Recently, *Arabidopsis Pseudo*-*Response Regulator2* (*APRR2*) was identified as the candidate gene controlling peel color in wax gourds [6].

Auxin is the first identified plant hormone, and controls numerous aspects of plant growth and development by regulating cell division, expansion, differentiation, and patterning [10,11]. Auxin can induce the expression of various genes [12,13]. Currently, three major classes of early auxin-responsive gene families have been identified in plants, including the *Auxin*/*Indole-3-Acetic Acid* (*Aux*/*IAA*) family, the *Small Auxin Up-regulated RNA* (*SAUR*) family, and the *Gretchen Hagen3* (*GH3*) family [14], which play important roles in auxin signaling and homeostasis.

Among the early auxin-responsive gene families, the *SAUR* gene family is the largest [15,16]. In most plant genomes, the number of *SAUR* genes ranges from 60 to 140, and the genes are usually arranged in clusters [14,17,18,19]. The SAUR proteins are characterized by a conserved core of about 60 residues, named the SAUR domain [15,16]. *SAUR* genes were originally identified as a class of small RNAs that were rapidly induced by auxin in the soybean [18]. In the model plant *Arabidopsis*, the *SAUR* gene family contains 79 members and participates in a wide range of cellular, physiological, and developmental processes [15,16,20]. For example, *SAUR19*, *SAUR41*, and *SAUR63* promote hypocotyl elongation by increasing cell expansion [21,22,23]. PHYTOCHROME RAPIDLY REGULATED1 (PAR1) and PAR2 repress the expression of *SAUR15* and *SAUR68* to inhibit shade-avoidance responses [24]. The *SAUR19* subfamily genes function downstream of PHYTOCHROME INTERACTING FACTOR4 (PIF4) to regulate hypocotyl growth in response to high temperatures [25]. SAUR17 and SAUR50 proteins differentially regulate Protein Phosphatase 2C D-clade 1 (PP2C-D1) during apical hook development and cotyledon opening [26]. Moreover, the *SAUR19* subfamily genes positively regulate leaf growth [22], while *SAUR36* negatively regulates leaf cell expansion and positively regulates leaf senescence [27]. It was also shown that some SAUR proteins, such as SAUR19, SAUR41, and SAUR63, are capable of modulating IAA transport [21,22,23]. In addition to auxin, brassinosteroid (BR) [28], gibberellin (GA) [29], jasmonate (JA) [30], abscisic acid (ABA) [31], and cytokinin (CK) [32] also regulate the expression of some *SAUR* genes.

Furthermore, it has been claimed that the *SAUR* genes are also involved in fruit development and ripening. In *Arabidopsis*, overexpression of *SAUR8*, *SAUR10*, and *SAUR16* led to longer siliques when compared with the wild-type (WT) version [32,33]. Tomato *SlSAUR69* could repress auxin transport and enhance sensitivity to ethylene at the onset of fruit ripening [34]. In grapes, *SAUR* members were identified and *SAUR041* was proposed to be a candidate regulator of berry size [35]. In cucurbit crops, the *SAUR* gene family had been characterized in watermelon, cucumber, and melon [36,37,38], and some *SAUR* genes were significantly accumulated in the fruits, suggesting their potential roles in controlling fruit development.

Previously, we identified a *SAUR* gene as the candidate domestication gene conferring large fruit size in wax gourds [7], while no comprehensive analysis of the *SAUR* gene family has been reported in wax gourds. In this study, we performed genome-wide identification of the *SAUR* gene family in wax gourds and analyzed their syntenic and phylogenetic relationships. We determined their gene structures, conserved motifs, *cis*-acting elements, and expression patterns. Then, we selected *BhSAUR60* for further characterization. Our results provide systematic information on the wax gourd *SAUR* gene family, which will facilitate the study of functional and regulatory mechanisms of *SAUR* family members during wax gourd fruit development.

## 2. Results

### 2.1. Identification of the SAUR Gene Family in Wax Gourds

**Figure 1:**
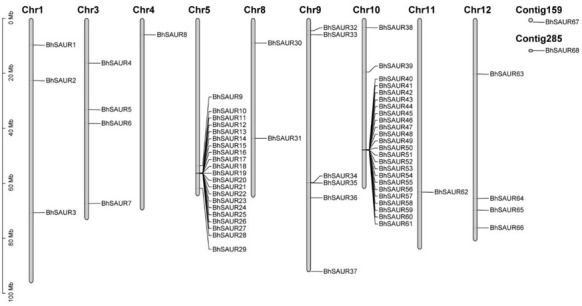
Chromosomal location of the *BhSAUR* genes.

To identify the *SAUR* gene family in wax gourds, HMMER searches were performed in the genome of wax gourd inbred line B227 using the *SAUR* gene family characteristic domain (Pfam: PF02519). A total of 68 *SAUR* genes were obtained (Table S1). A multiple sequence alignment analysis identified the conserved SAUR domain of BhSAUR proteins (Figure S1), which was consistent with the previous report [15]. According to their location on the chromosomes, the 68 wax gourd *SAUR* genes were named, from *BhSAUR1* to *BhSAUR68* (Figure 1). Among the 68 genes, 66 were distributed on the nine chromosomes, with *BhSAUR67* and *BhSAUR68* not mapped to the existing chromosomes (Figure 1). Moreover, *BhSAUR10*–*BhSAUR28* and *BhSAUR40*–*BhSAUR61* constituted two gene clusters on chromosome 5 and chromosome 10, respectively (Figure 1).

The open reading frame (ORF) length of the 68 *BhSAUR* genes ranged from 225 to 573 bp, encoding polypeptides of 74–190 amino acids (aa) (Table S1). The molecular weight (Mw) and theoretical isoelectric point (pI) of their proteins were 8.27–22.29 kDa and 4.60–10.67, respectively (Table S1). Subcellular localization prediction results showed that most BhSAUR proteins were located in the chloroplast or nucleus, and only a few of them were located in the mitochondrion, cytoplasm, cell membrane, or peroxisome (Table S1).

### 2.2. Gene Duplication and Synteny Analysis of the BhSAUR Gene Family 

As the *SAUR* gene family is a large family, we analyzed the gene duplication modes of *BhSAUR* genes. We found that *BhSAUR* genes were derived from five types of duplication events, including whole-genome duplication (WGD), tandem duplication (TD), dispersed duplication (DSD), proximal duplication (PD), and transposed duplication (TRD) (Table S2). Most genes (36 members) were derived from TD (Table S2), indicating that the TD event was the main driver of *SAUR* gene family expansion in wax gourds. In addition, all tandem-duplicated genes were located in the two *SAUR* gene clusters.

**Figure 2:**
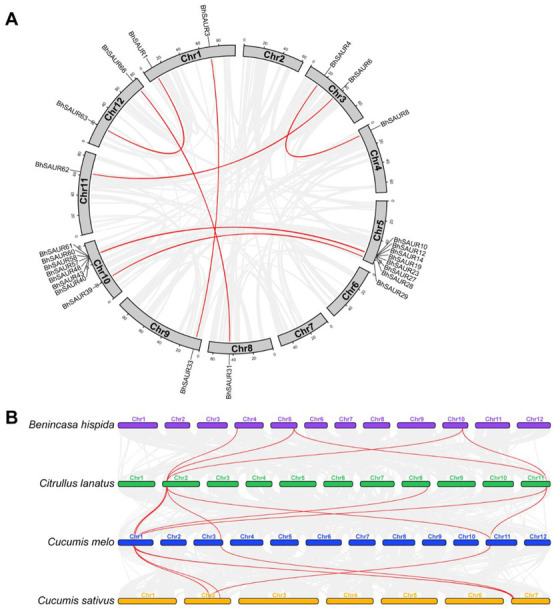
Synteny analysis of the *SAUR* genes. (**A**) Synteny analysis of the *SAUR* genes in wax gourds. Grey lines display the collinear gene pairs in the wax gourd genome. Red lines indicate the collinear *BhSAUR* gene pairs; (**B**) gene synteny analysis across the wax gourd (*Benincasa hispida*), watermelon (*Citrullus lanatus*), melon (*Cucumis melo*), and cucumber (*Cucumis sativus*). Grey lines display the collinear gene pairs across four cucurbit crop genomes. Red lines indicate the collinear *SAUR* gene pairs in the gene clusters.

To detect and evolutionarily analyze the synteny of *BhSAUR* genes, we performed an intra-species synteny analysis of the wax gourd genome. Finally, 13 paralogous *BhSAUR* gene pairs were identified, among which seven *BhSAUR* gene pairs were found between the two gene clusters (Figure 2A). In addition, we calculated the non-synonymous substitutions/synonymous substitutions (Ka/Ks) values of the *BhSAUR* gene pairs to evaluate sequence variations, and all Ka/Ks values were below 1 (Table S3), indicating that these genes had undergone purifying selection throughout their evolution.

The *SAUR* gene family had been identified in other cucurbit crops, including watermelon, melon, and cucumber, which also contained two *SAUR* gene clusters [36,37,38]. To explore the evolutionary trajectory of the *SAUR* family, we constructed a syntenic map and generated collinear *SAUR* gene pairs across four cucurbit crops (Figure S2; Table S4), which showed that most *SAUR* genes were mapped to these syntenic blocks (Figure S2). Furthermore, we analyzed the collinear gene pairs in the *SAUR* gene clusters. The obtained results showed that genes in the *SAUR* gene clusters displayed one-to-one, one-to-many, or many-to-one syntenic relationships between any two cucurbit crops (Figure 2B).

### 2.3. Phylogenetic Analysis of the BhSAUR Gene Family

**Figure 3:**
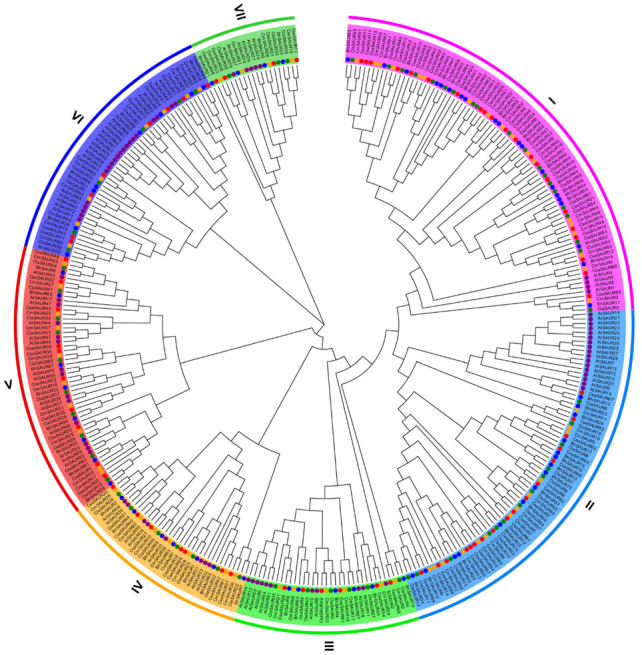
Phylogenetic tree of the SAUR family. The unrooted tree was constructed from all SAUR proteins of *Arabidopsis* (At), wax gourd (Bh), watermelon (Cla), melon (Cm), and cucumber (Csa). Different color circles represent different species. I–VII represent the seven subfamilies.

To understand the phylogenetic relationships among the identified SAUR proteins in cucurbit crops, we constructed a neighbor-joining phylogenetic tree using the complete amino acid sequences of SAURs from the wax gourd, watermelon, melon, cucumber, and *Arabidopsis*. The obtained phylogenetic tree showed that these SAURs were divided into seven subfamilies (Figure 3).

The SAUR proteins were randomly grouped into their subfamilies except for subfamilies I and II, as the SAUR proteins of cucurbit crops were branched together while the *Arabidopsis* SAUR proteins were branched together in these two subfamilies (Figure 3). In addition, most clustered BhSAUR proteins on chromosome 10 and chromosome 5 were classified into subfamily I and subfamily II, respectively (Figure 3).

### 2.4. Gene Structure and Conserved Motif Analysis of the BhSAUR Gene Family

**Figure 4:**
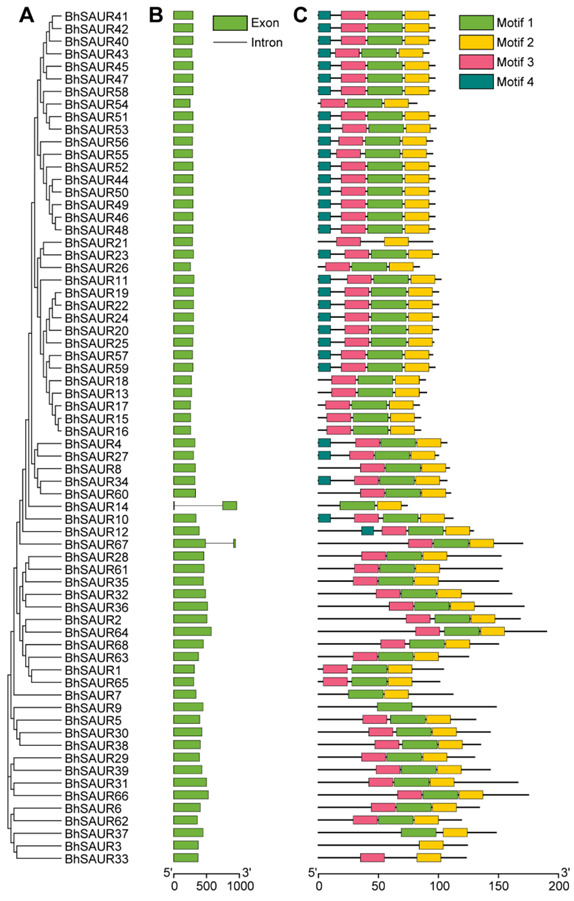
Gene structure and conserved motifs of the *BhSAUR* genes. (**A**) Phylogenetic tree of the BhSAUR proteins; (**B**) gene structure of the *BhSAUR* genes; (**C**) conserved motifs of the BhSAUR proteins.

To investigate the exon and intron structures of *BhSAUR* genes, their coding sequences and genomic sequences were extracted for comparison. The obtained results showed that most *BhSAUR* genes did not contain introns, and only two genes (*BhSAUR14* and *BhSAUR67*) contained one intron (Figure 4B). We then used the MEME online software to analyze the conserved motifs of BhSAUR proteins. Four conserved motifs were identified, and the closely related BhSAUR proteins had similar motifs (Figure 4A,C). Motifs 1–3 constituted the SAUR domain, and 61 BhSAUR proteins contained all three of these motifs, while the other seven contained only one or two motifs (Figure 4C). In addition, motif 4 was present only in some BhSAUR proteins (Figure 4C), most of which were in the two gene clusters on chromosome 5 and chromosome 10, indicating that motif 4 may contribute to the special function of these proteins.

### 2.5. Cis-Acting Element Analysis in the Promoters of the BhSAUR Gene Family

**Figure 5:**
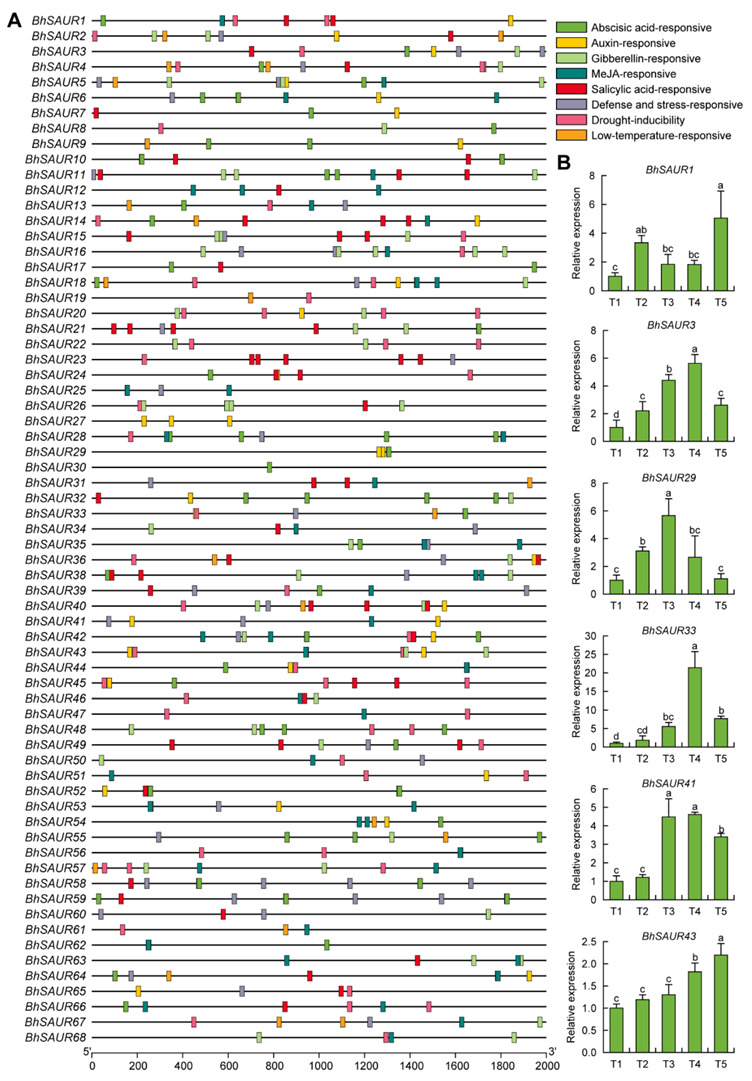
*Cis*-acting element analysis of the *BhSAUR* gene promoters. (**A**) *Cis*-acting elements in the promoters of the *BhSAUR* genes; (**B**) expression analysis of the *BhSAUR* genes under IAA treatment. T1, T2, T3, T4, and T5 represent 0, 10, 30, 60, and 120 min after IAA treatment, respectively. Values are means ± SDs (*n* = 3). Different lowercase letters indicate significant differences determined by one-way ANOVA with Duncan’s test (*p* < 0.05).

To identify the *cis*-acting elements in the promoters of *BhSAUR* genes, their 2000 bp promoter regions upstream of the start codon were obtained and analyzed with the PlantCARE online software. Almost all *BhSAUR* gene promoters contained one or multiple *cis*-acting elements involved in plant hormone responses (Figure 5A), including abscisic acid-responsive element (ABRE), auxin-responsive elements (AuxRE, TGA-element, AuxRR-core), gibberellin-responsive elements (GARE-motif, P-box, TATC-box), methyl jasmonate (MeJA)-responsive elements (CGTCA-motif, TGACG-motif), and salicylic acid-responsive element (TCA-element). In addition, *cis*-acting elements associated with environmental signal response were also found (Figure 5A), such as a defense and stress-responsive element (TC-rich repeats), drought-inducibility element (MBS), and low-temperature-responsive element (LTR). Therefore, plant hormones and environmental conditions might be important for regulating the expression of *BhSAUR* genes.

As *SAUR* genes are major early auxin-responsive genes and putative auxin-responsive *cis*-acting elements were found in some *BhSAUR* gene promoters, we performed an IAA-treatment experiment to test whether these genes could respond to auxin. Then, six *BhSAUR* genes with auxin-responsive *cis*-acting elements in their promoters were randomly selected for validation. Quantitative real-time PCR (qRT-PCR) results showed that all selected genes responded to IAA treatment (Figure 5B). *BhSAUR1*, *BhSAUR3*, and *BhSAUR29* were upregulated at 10 min after IAA treatment, while the expression of the other three genes increased significantly at 30 or 60 min (Figure 5B). Moreover, the expression of *BhSAUR3*, *BhSAUR29*, *BhSAUR33*, and *BhSAUR41* decreased at 60 or 120 min (Figure 5B). Our results implied that these *BhSAUR* genes were likely to play critical roles in the IAA signaling transduction pathway.

### 2.6. Expression Characterization of the BhSAUR Gene Family

**Figure 6:**
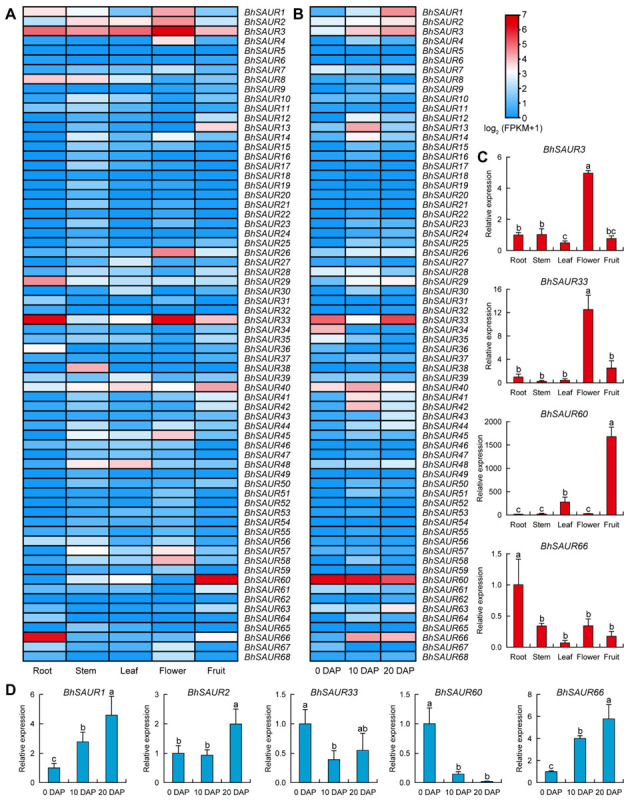
Expression characterization of the *BhSAUR* genes. (**A**) Expression profile of the *BhSAUR* genes in different tissues; (**B**) expression profile of the *BhSAUR* genes in fruits at different developmental stages. DAP, days after pollination; (**C**) validation of the expression of some *BhSAUR* genes in different tissues. Values are means ± SDs (*n* = 3); (**D**) validation of the expression of some *BhSAUR* genes in fruits at different developmental stages. Values are means ± SDs (*n* = 3). Different lowercase letters in (**C**,**D**) indicate significant differences determined by one-way ANOVA with Duncan’s test (*p* < 0.05).

To further explore the function of *BhSAUR* genes, we investigated their expression patterns through previously published transcriptome data [7,39]. Expression profiles of the *BhSAUR* genes in different tissues showed that these genes displayed different expression patterns (Figure 6A). For example, *BhSAUR3* was highly expressed in every tissue, with higher expression in the flower. *BhSAUR33* showed higher expression in the root and flower than in other tissues. *BhSAUR60* and *BhSAUR66* were specially expressed in the fruit and root, respectively. Moreover, *BhSAUR60* exhibited the highest expression in the fruit among the 68 *BhSAUR* genes. We then performed a qRT-PCR analysis to validate the expression patterns of these four genes, and the results were generally consistent with the transcriptome data (Figure 6C).

In addition, we analyzed the expression profiles of *BhSAUR* genes in fruits at different developmental stages and found that several of them were expressed at one to three stages (Figure 6B). Our qRT-PCR results also verified the expression patterns of five *BhSAUR* genes (Figure 6D). Consistently, the expression of *BhSAUR1*, *BhSAUR2*, and *BhSAUR66* increased with the development of fruit. *BhSAUR33* showed higher expression in 0- and 20-days after pollination (DAP) fruits than in 10-DAP fruits. *BhSAUR60* was highly expressed in 0-DAP fruits, and its expression decreased as the fruit developed. Together, these analyses suggested that *BhSAUR60* might be crucial in wax gourd fruit development, and we chose *BhSAUR60* for further characterization.

### 2.7. Subcellular Localization Analysis of the BhSAUR60 Protein

**Figure 7:**
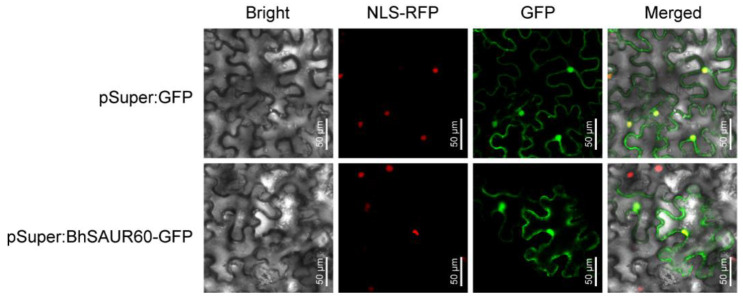
Subcellular localization of the BhSAUR60 Protein.

To determine the subcellular localization of BhSAUR60 protein, the green fluorescent protein (GFP) tag was fused to the C terminus of *BhSAUR60* under the control of the super promoter. The control construct and *Super:BhSAUR60–GFP* recombinant construct were transferred into *Nicotiana benthamiana* leaves using *Agrobacterium tumefaciens*-mediated transient expression. The result showed that the BhSAUR60–GFP fusion protein was localized to both the nucleus and cytoplasm (Figure 7).

### 2.8. Functional Analysis of the BhSAUR60 Gene

**Figure 8:**
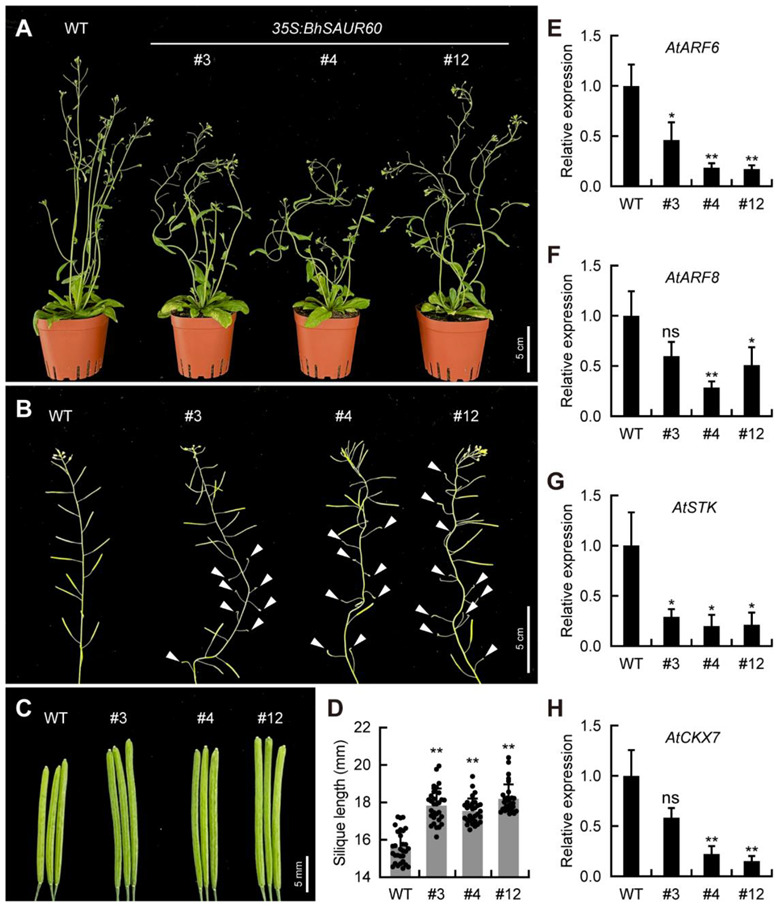
Overexpression of the *BhSAUR60* gene in *Arabidopsis*. (**A**) Whole-plant phenotypes of wild-type (WT) and *35S:BhSAUR60* plants around 10 days after bolting (DAB); (**B**) main-stem phenotypes of WT and *35S:BhSAUR60* plants at 18 DAB. Triangles indicate unfertilized pistils; (**C**) mature siliques of WT and *35S:BhSAUR60* plants; (**D**) silique lengths of WT and *35S:BhSAUR60* plants. Values are means ± SDs (*n* = 30), **, *p* < 0.01 (Student’s *t*-test); (**E**–**H**) expression analysis of fruit development-related genes in WT and *35S:BhSAUR60* siliques. Values are means ± SDs (*n* = 3), ns, not significant, *, *p* < 0.05, **, *p* < 0.01 (Student’s *t*-test).

Phylogenetic analysis showed that BhSAUR60 was closely related to the *Arabidopsis* SAUR10 (AtSAUR10) protein and grouped into the subfamily III (Figure 3), indicating that BhSAUR60 may have similar functions as AtSAUR10. In *Arabidopsis*, overexpression of *AtSAUR10* showed pleiotropic growth-related phenotypes, including longer hypocotyls, cauline leaves, sepals, filaments, pistils, and siliques, and wavier stems [32,33]. To further investigate the biological function of *BhSAUR60*, we generated a *35S:BhSAUR60* construct and transformed it into *Arabidopsis*. All transgenic lines showed similar phenotypes, and we selected three representative lines for further characterization (Figure 8A). The expression of *BhSAUR60* increased significantly in the transgenic lines (Figure S3). Overexpression of *BhSAUR60* in *Arabidopsis* displayed wavy main stems and side branches (Figure 8A,B). The overexpression lines also displayed longer floral organs, including longer sepals, filaments, and pistils (Figure S4). Due to the long pistil size, the overexpression lines often showed reduced fertility, and long siliques were only reached in pollinated pistils (Figure 8B). Measurement of the well-developed siliques showed that the silique lengths of overexpression lines were significantly increased when compared with those of WT (Figure 8C,D). These results indicated that *BhSAUR60* could promote fruit elongation.

Furthermore, we performed an expression analysis of fruit development-related genes in WT and *35S:BhSAUR60* siliques. Our qRT-PCR results showed that the expression levels of *AtARF6*, *AtARF8*, *AtSTK*, and *AtCKX7* decreased significantly in the *35S:BhSAUR60* plants (Figure 8E–H). These genes regulate auxin signaling or cytokinin degradation to influence fruit elongation in *Arabidopsis* [40,41], which implied that *BhSAUR60* might regulate fruit elongation of *Arabidopsis* through the auxin and cytokinin pathways.

## 3. Discussion

The *SAUR* family is the largest family of early auxin-responsive genes and participates in a variety of processes of plant growth and development [15,16]. Previously, *SAUR* genes have been identified in the model plant *Arabidopsis* and other diverse plant species [15,20]. In cucurbit crops, there are 65, 73, and 66 *SAUR* genes in watermelon [37], cucumber [36], and melon [38], respectively. In this study, we identified 68 *SAUR* genes in the wax gourd genome (Figure 1; Table S1), a number of *SAURs* similar to those of cucurbit crops. Together with evolutionary relationships (Figure 2 and Figure 3), bioinformatic analysis (Figure 4 and Figure 5), and expression profiles (Figure 6), the comprehensive characteristics of *BhSAUR* genes were also discovered.

The 68 *BhSAUR* genes identified were distributed on the nine chromosomes and two contigs, with *BhSAUR10*–*BhSAUR28* and *BhSAUR40*–*BhSAUR61* constituting two gene clusters on chromosomes 5 and chromosomes 10, respectively (Figure 1). Previous studies indicated that tandem duplicates have been found to play significant roles in plant adaptations to rapidly changing environments [42,43]. Our results showed that most *BhSAUR* genes were derived from TD events (Table S2), and all tandem-duplicated genes were located in the two *SAUR* gene clusters (Figure 1), indicating that TD events led to the expansion of the *SAUR* gene family and increased gene diversity in the wax gourd.

In cucurbit crops, the basic helix–loop–helix (bHLH) transcription factor family had over 100 members, and over 10 orthologous *bHLH* genes formed a superblock and had perfect syntenic relationships among seven cucurbit crop genomes [44]. There were two *SAUR* gene clusters in the wax gourd, watermelon, melon, and cucumber [36,37,38]. In this study, we detected the syntenic *SAUR* genes across four cucurbit crops (Table S4), while no perfectly matched syntenic block was found in the *SAUR* clusters across four cucurbit crops (Figure 2B). Therefore, we speculate that the conserved syntenic block across cucurbit crops may be lost due to gene duplication during the evolution and divergence of *SAUR* genes.

It was shown that *SAUR* genes integrate hormonal and environmental signals to regulate plant growth and development [15,16]. In the present study, we identified *cis*-acting elements associated with plant hormone responses in the *BhSAUR* gene promoters, including ABA, auxin, GA, MeJA, and salicylic acid responses (Figure 5A). Additionally, *cis*-acting elements in response to environmental signals were also found, such as defense- and stress-responsive elements, drought-inducibility elements, and low-temperature-responsive elements (Figure 5B). These results indicate that plant hormones and environmental signals may be important for regulating the expression of *BhSAUR* genes. As *SAUR* genes comprise the largest family of early auxin-responsive genes, we performed an IAA-treatment experiment to test whether these genes could respond to auxin. The obtained results showed that selected *BhSAUR* genes, including *BhSAUR1*, *BhSAUR3*, *BhSAUR29*, *BhSAUR33*, *BhSAUR41*, and *BhSAUR43* responded to auxin (Figure 5C).

Phylogenetic analysis of the SAUR proteins from four cucurbit crops and *Arabidopsis* showed that these SAURs were classified into seven subfamilies (Figure 3). In addition, most of the clustered BhSAUR proteins on chromosomes 10 and 5 were classified into subfamilies I and II, respectively (Figure 3). Although *BhSAUR60* was a member of the *BhSAUR* gene cluster on chromosome 10, we found that the BhSAUR60 protein lacked motif 4 and did not group with the clustered BhSAUR proteins into subfamily I (Figure 3 and Figure 4), which implies that BhSAUR60 may have distinct functions from these proteins. In *Arabidopsis*, eight closely related SAUR proteins constitute the SAUR10-clade, and their overexpression lines all showed similar growth-related phenotypes, including longer hypocotyls, cauline leaves, sepals, filaments, pistils, and siliques, as well as wavy stems [32,33]. Our phylogenetic analysis showed that BhSAUR60 was more closely related to the members of *Arabidopsis* SAUR10-clade in the subfamily III (Figure 3), and overexpression of *BhSAUR60* in *Arabidopsis* also displayed wavy stems and longer organs (Figure 8A–C; Figure S4), suggesting that it had the conserved functions of the SAUR10-clade proteins in *Arabidopsis*, especially the regulating of cell elongation. Previously, we also demonstrated the function of BhSAUR60 in cell expansion in wax gourds [7]. Together, these results imply that BhSAUR60 may have the ability to promote fruit elongation in the wax gourd.

Fruits are the main, edible part of cucurbits, which have diverse fruit sizes and shapes [2,45]. Fruit size affects the appearance quality and the yield of wax gourds, while the molecular mechanisms regulating fruit development remain largely unknown [7]. In this study, we analyzed the expression profiles of *BhSAUR* genes using previously published transcriptome data, and found that several of them were expressed in fruits at one to three stages (Figure 6A,B). Previously, a study had identified several regions/genes potentially selected during wax gourd domestication, and *Bhi10G001538* (*BhSAUR60* in this study) was proposed to be an important candidate domestication gene conferring large fruit size in the wax gourd [7]. In the present study, we demonstrated that *BhSAUR60* was highly expressed in the fruits specifically (Figure 6C,D), and that overexpression led to longer fruits in *Arabidopsis* (Figure 8C,D), suggesting the crucial role of *BhSAUR60* during fruit development. 

## 4. Materials and Methods

### 4.1. Plant Materials and Treatment

Wax gourd inbred lines B227 (reference genome-based variety) and P109 were used as the experimental materials in this study. The original seeds were provided by the Vegetable Research Institute of the Guangdong Academy of Agricultural Sciences. The B227 variety was grown in the research-experimental field of the Vegetable Research Institute, Guangdong Academy of Agricultural Sciences under the natural sunlight conditions with standard agricultural practices. Roots, stems, leaves, male flowers (indicated as flowers), and fruits of the B227 variety were collected with three biological replicates at 0 DAP, 10 DAP, and 20 DAP. For auxin treatment, the P109 variety was grown in a greenhouse of the Vegetable Research Institute, Guangdong Academy of Agricultural Sciences under a cycle of 16-h light and 8-h dark at 25 °C. The fully expanded leaves of 3-week-old seedlings were sprayed with 100 μM IAA, and leaf samples were collected at 0, 10, 30, 60, and 120 min after treatment with three biological replicates. All samples were immediately frozen in liquid nitrogen and stored at −80 °C for subsequent use.

### 4.2. Identification of the Wax Gourd SAUR Gene Family

The SAUR proteins of wax gourds were identified from the B227 genome database using the HMMER software (version 3.3.2, Howard Hughes Medical Institute, Chevy Chase, MD, USA) based on the SAUR Hidden Markov Model (HMM) profile from the Pfam database (PF02519, the characteristic domain of auxin-inducible *SAUR* gene family) [46,47]. The identified BhSAUR proteins with an E-value of less than 10^−5^ were reserved for further analysis. Multiple sequence alignment of BhSAUR proteins was performed on WebLogo [48], and the sequence logo of the SAUR domain was then generated. The chromosomal location of the *BhSAUR* genes was visualized using the TBtools software (version 1.0987663, South China Agricultural University, Guangzhou, China) based on the gene annotation file of the wax gourd [49]. The molecular weight, theoretical isoelectric point, and other physical and chemical properties of BhSAUR proteins were analyzed with the ExPASY Server [50]. The subcellular localization of BhSAUR proteins was predicted by the Plant-mPLoc [51]. The basic information of *BhSAUR* genes is shown in Table S1.

### 4.3. Gene Duplication and Synteny Analysis

DupGen_finder was used to identify the different modes of gene duplication in wax gourds [42]. Gene duplication modes of *BhSAUR* genes are listed in Table S2. Gene synteny analysis was performed using the *MCScanX* toolkit with default parameters [52]. Based on the syntenic blocks, intra-species paralogous genes and inter-species orthologous genes were identified. Paralogous gene pairs and orthologous gene pairs are listed in Table S3 and Table S4, respectively. Then, the orthologous gene pairs among four cucurbit crops were merged. Visualization of collinear gene pairs was performed using the Advanced Circos or Multiple Synteny Plot function of TBtools software (version 1.0987663) [49]. The non-synonymous substitutions (Ka), synonymous substitutions (Ks), and Ka/Ks values of *BhSAUR* paralogous gene pairs were calculated by the KaKs Calculator and are listed in Table S3 [53].

### 4.4. Phylogenetic Analysis

Sequences of identified SAUR proteins in the wax gourd, watermelon, melon, and cucumber were obtained from the Cucurbit Genomics Database (http://cucurbitgenomics.org/, accessed on 14 December 2021). Sequences of *Arabidopsis* SAUR proteins were obtained from the Arabidopsis Information Resource (https://www.arabidopsis.org, accessed on 14 December 2021). Alignment of all SAUR protein sequences was carried out using the ClustalW tool in the MEGA X software (version 10.2.6, The Pennsylvania State University, University Park, PA, USA) [54]. Based on the results of multiple sequence alignment, the phylogenetic tree was constructed by the MEGA X software (version 10.2.6) using the neighbor-joining method with 1000 bootstrap value and 50% partial deletion for gap treatment [54]. Then, the resulting phylogeny was visualized using the Evolview tool [55]. The accession numbers of genes are listed in Table S5.

### 4.5. Gene Structure Analysis and Conserved Motif Prediction

Comparison of the gene structure was carried out using the Gene Structure Display Server [56]. To predict the conserved motifs of BhSAUR proteins, the complete amino acid sequences of BhSAURs were analyzed using the MEME Suite (https://meme-suite.org/meme/meme_5.4.1/tools/meme, accessed on 22 June 2022) [57]. The number of motifs was set to 4, and the other parameters were default. Visualization of gene structure and conserved motifs was performed using the TBtools software (version 1.0987663) [49].

### 4.6. Cis-Acting Element Analysis

The promoter sequences (2000 bp genomic sequence upstream of the start codon) of the *BhSAUR* genes were extracted from the wax gourd genome database. *Cis*-acting elements of the promoter regions were analyzed using the PlantCARE online software (https://bioinformatics.psb.ugent.be/webtools/plantcare/html/, accessed on 20 May 2022) [58]. Visualization of *cis*-acting elements was performed using the TBtools software (version 1.0987663) [49].

### 4.7. Expression Profile Analysis

The transcriptome data of five wax gourd tissues (root, stem, leaf, flower, and fruit) and fruits at three developmental stages (0 DAP, 10 DAP, and 20 DAP) were obtained from previously published studies [7,39]. The average fragments per kilobase of transcript per million mapped reads (FPKM) values of each gene in different samples were used for further expression analysis. Heatmaps of the expression patterns of *BhSAUR* genes were created using the TBtools software (version 1.0987663) [49].

### 4.8. RNA Extraction and qRT-PCR

Total RNA of different tissues was extracted using the *TransZol* Up Plus RNA Kit (TransGen Biotech, Beijing, China). Then, a FastKing gDNA Dispelling RT SuperMix Kit (Tiangen Biotech, Beijing, China) was used to synthesize the cDNAs. The qRT-PCR was performed on the CFX96™ Real-Time PCR Detection System (Bio-Rad, Hercules, CA, USA) using the *PerfectStart*^®^ Green qPCR SuperMix (TransGen Biotech, Beijing, China). All reactions were performed with three biological replicates and three technical replicates. The wax gourd ubiquitin gene (*Bhi10G000739*) and *Arabidopsis* actin gene (*AT5G09810*) were used as internal references. The relative expression levels were calculated by the 2^−ΔΔCT^ method [59]. All primers specific to the templates were designed using the NCBI Primer-BLAST tool. Primers used for qRT-PCR are listed in Table S6.

### 4.9. Subcellular Localization Analysis

The full-length coding sequence (CDS), without the stop codon of *BhSAUR60,* was cloned and inserted into the pSuper-1300 GFP vector to fuse with the GFP tag. The recombinant construct and empty vector were transformed into *A. tumefaciens* strain GV3101. The *N. benthamiana* plants were grown in a greenhouse under the 16-h light/8-h dark cycle at 25 °C for five weeks. Then, bacterial suspensions were infiltrated into fully expanded young leaves of *N. benthamiana* plants. The RFP-labeled nuclear marker (NLS-RFP) was used to mark the nucleus position. After 2 d of infiltration, fluorescence signals of the leaf epidermal cells were visualized using a confocal laser scanning microscope (Zeiss LSM710, Jena, Germany). Primers used for vector construction are listed in Table S6.

### 4.10. Transformation of Arabidopsis and Trait Measurement

The CDS of *BhSAUR60* was inserted into the pBI121 vector under the control of the 35S promoter. The recombinant construct was transformed into *A. tumefaciens* strain GV3101. *Arabidopsis* Columbia ecotype (Col-0) was used as the WT. The *Arabidopsis* plants were grown in a growth chamber under the 16-h light/8-h dark cycle at 22 °C. *Agrobacterium*-mediated transformation of *Arabidopsis* was performed using the floral dip method [60]. Transgenic plants were selected by kanamycin resistance and further confirmed by PCR. T_2_-generation plants were used for functional identification. The silique length (from stigma to gynophore) of three individual plants was measured for each line, with ten well-developed siliques from the middle of the main inflorescence per individual plant. Finally, the average length of 30 siliques was measured to represent each transgenic line. Primers used for vector construction and genotyping are listed in Table S6.

## 5. Conclusions

In the present study, we identified 68 *SAUR* genes in the wax gourd, which were distributed on nine chromosomes with 41 genes in two clusters. More than half of the *BhSAUR* genes were derived from tandem-duplication events. The BhSAUR proteins were classified into seven subfamilies, and members grouped into the same subfamily shared similar conserved motifs and arrangements. The *BhSAUR* gene promoters contained *cis*-acting elements involved in plant hormone responses, including the ABA, auxin, GA, MeJA, and salicylic acid responses. The IAA-treatment experiment and qRT-PCR analysis showed that *BhSAUR1*, *BhSAUR3*, *BhSAUR29*, *BhSAUR33*, *BhSAUR41*, and *BhSAUR43* could respond to auxin. In addition, *cis*-acting elements in response to environmental signals, such as defense- and stress-responsive elements, drought-inducibility elements, and low-temperature-responsive elements, were also found in the *BhSAUR* gene promoters. Further expression profiles showed that *BhSAUR* genes displayed different expression patterns. *BhSAUR3*, *BhSAUR60,* and *BhSAUR66* were predominantly expressed in the flower, fruit, and root, respectively. Furthermore, we demonstrated that the BhSAUR60 protein was localized to the nucleus and cytoplasm, and that its overexpression led to increased fruit length in *Arabidopsis*. These results enhanced our understanding of the *SAUR* gene family in wax gourds and revealed the potential role of *BhSAUR60* in regulating fruit elongation. In future studies, the detailed functions of *BhSAUR60* will be elucidated through genome editing and overexpression technologies, thereby revealing the regulatory mechanisms of *BhSAUR60* in determining fruit length and facilitating the molecular breeding of wax gourds.

## Data Availability

The sequence data of the wax gourd, watermelon, melon, and cucumber can be found in the Cucurbit Genomics Database (http://cucurbitgenomics.org/, accessed on 14 December 2021) under accession numbers in Tables S1 and S5. The sequence data of *Arabidopsis* can be found in the Arabidopsis Information Resource (https://www.arabidopsis.org, accessed on 14 December 2021) under accession numbers in Table S5. Transcriptome data of the wax gourd were downloaded from the National Center for Biotechnology Information (NCBI) SRA database under accession number SRP224600 and SRA074508.

## Supplementary Materials

The following supporting information can be downloaded at: https://www.mdpi.com/article/10.3390/ijms232214021/s1.
