# Supplementary material for: Genome-Wide Identification of the SAUR Gene Family in Wax Gourd (Benincasa hispida) and Functional Characterization of BhSAUR60 during Fruit Development
Source: Int J Mol Sci. 2022 Nov 14;23(22):14021. doi: 10.3390/ijms232214021 (PMC9694812; doi:10.3390/ijms232214021)
Supplement: Supplementary file 1 [file ijms-23-14021-s001.zip › BhSAUR-Supplementary Figures.pdf]

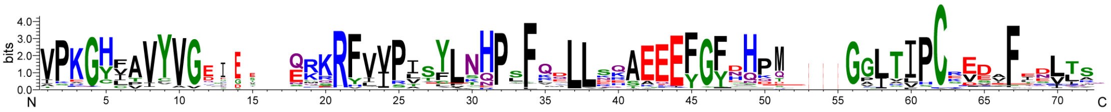

Figure S1. Sequence logo of the SAUR domain of BhSAUR proteins. The consensus residue(s) at each position are color coded.

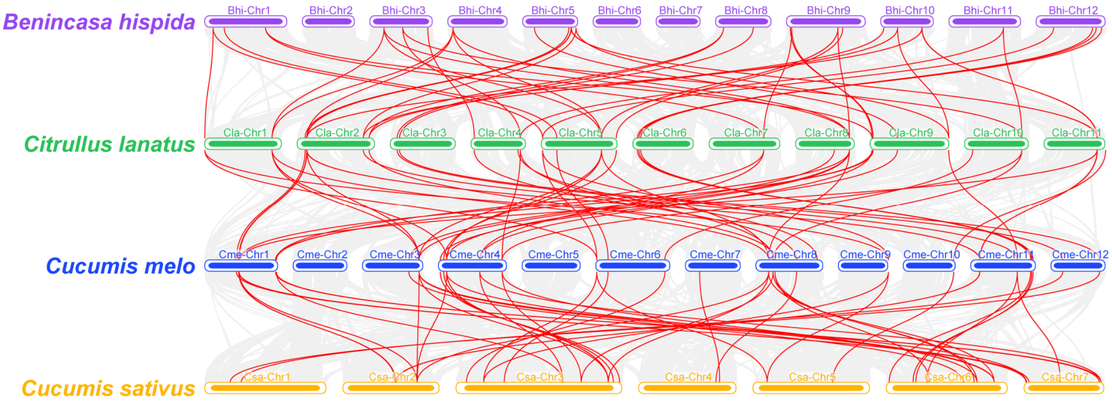

Figure S2. Gene synteny analysis among four cucurbit crop genomes. Grey lines display the collinear gene pairs among wax gourd (*Benincasa hispida*, Bhi), watermelon (*Citrullus lanatus*, Cla), melon (*Cucumis melo*, Cme), and cucumber (*Cucumis sativus*, Csa). Red lines indicate the collinear *SAUR* gene pairs.

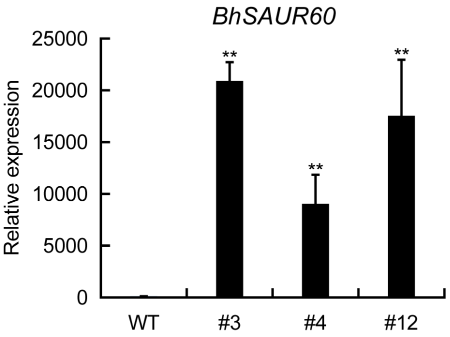

Figure S3. Expression of *BhSAUR60* in WT and *35S:BhSAUR60* siliques. Values are means ± SDs (n = 3); **, $p < 0.01$ (Student's t-test).

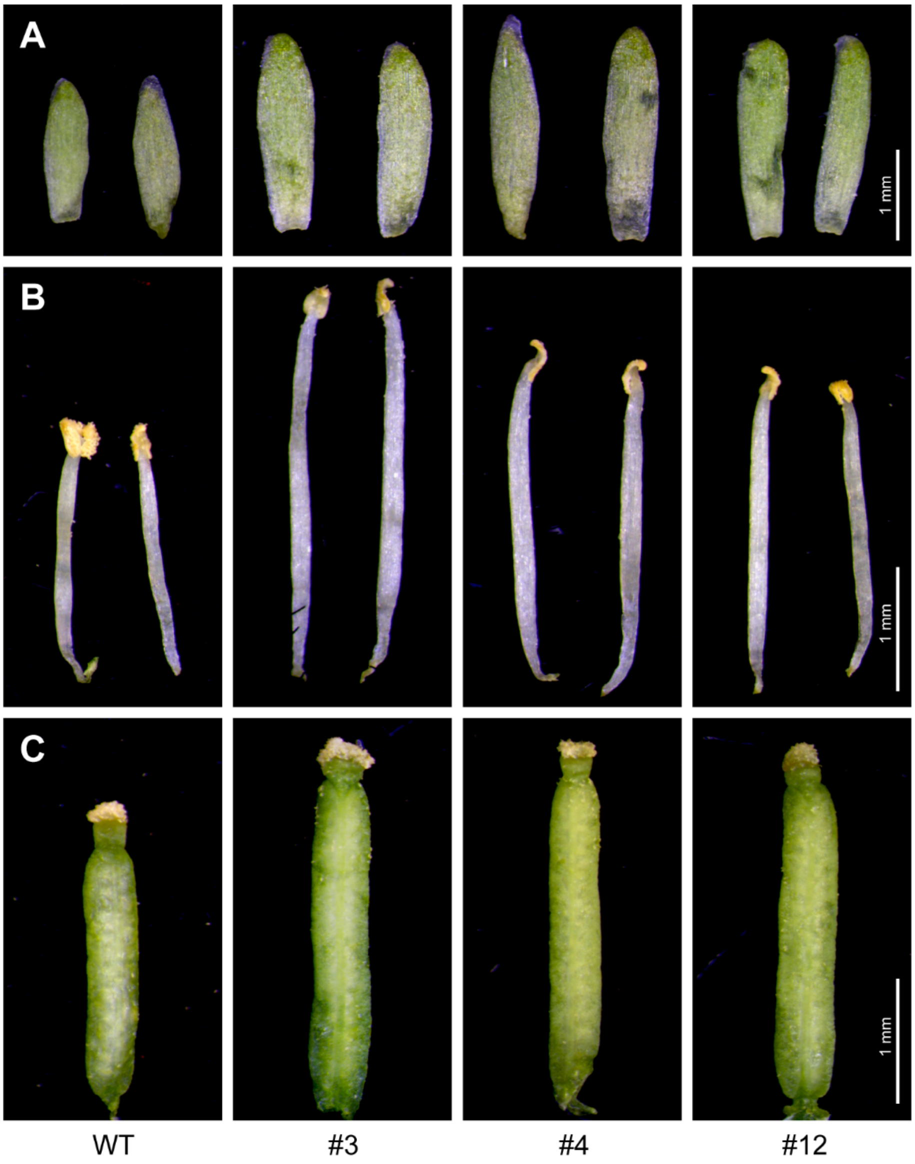

Figure S4. Overexpression of *BhSAUR60* in *Arabidopsis* resulted in longer floral organs. (**A**) Sepal phenotypes of 0 days after pollination (DAP) flowers; (**B**) filament phenotypes of 0 DAP flowers; (**C**) pistil phenotypes of 0 DAP flowers.
